# Cognitive limitations in depth estimation for dispatcher-assisted cardiopulmonary resuscitation: a prospective simulation study

**DOI:** 10.1016/j.resplu.2025.101093

**Published:** 2025-09-11

**Authors:** Min Woo Kim, Stephen Gyung Won Lee, Tae Han Kim, Yoon Ha Joo, Ki Jeong Hong

**Affiliations:** a Department of Emergency Medicine, Seoul National University Hospital; b Laboratory of Emergency Medical Services, Seoul National University Hospital Biomedical Research Institute; c Department of Emergency Medicine, Yonsei University College of Medicine; d Yonsei University Severance Hospital; e Department of Emergency Medicine, Seoul Metropolitan Government Seoul National University Boramae Medical Center

**Keywords:** Cardiopulmonary resuscitation, Out-of-hospital cardiac arrest, Dispatcher assisted cardiopulmonary resuscitation, Telephone cardiopulmonary resuscitation, Depth perception

## Abstract

**Background:**

Dispatcher-assisted cardiopulmonary resuscitation (DACPR) protocols often instruct bystanders to perform chest compressions to a target depth of 50–60 mm. However, whether laypersons can accurately perceive and achieve these depth targets remains unclear. This study evaluated laypersons’ ability to estimate 50 mm in horizontal length, vertical depth, and chest compression depth.

**Methods:**

We conducted a prospective simulation study enrolling adult laypersons without cardiopulmonary resuscitation (CPR) training within two years. Participants were asked to draw a 50 mm line, press a vertical measurement plate to an estimated depth of 50 mm, and perform chest compressions to 50 mm. Tasks were repeated after provision of a 50 mm visual reference. Accuracy was assessed by calculating the mean difference from 50 mm and the proportion of estimations within the acceptable range (45–55 mm).

**Results:**

100 participants were enrolled. Horizontal length was significantly underestimated (mean difference −3.5 ± 16.6 mm, *p* = 0.036), with 25.0 % (95 % confidence interval [CI], 16.9 %–34.7 %) within the acceptable range. Vertical depth was significantly overestimated (mean difference +4.9 ± 19.4 mm, *p* = 0.044), with 26.0 % (95 % CI, 17.7 %–35.7 %) within range. Chest compression depth was significantly underestimated both before (44.5 ± 10.7 mm) and after (44.7 ± 11.0 mm) provision of visual reference (both *p* < 0.001), with no significant improvement after reference exposure (*p* = 0.548).

**Conclusion:**

Laypersons have significant difficulty estimating 50 mm in length, vertical depth, and applying target chest compression depth. Providing a visual reference did not significantly improve performance.

## Background

Out-of-hospital cardiac arrest (OHCA) is a major public health concern with a high mortality rate.^1^ Early and high-quality bystander cardiopulmonary resuscitation (CPR) is essential for improving survival outcomes in OHCA and the quality of chest compressions, particularly chest compression depth, significantly influences outcomes.^2^, ^3^, ^4^ Current international CPR guidelines recommend that chest compressions be performed to a depth of at least 50 mm but not exceeding 60 mm.^5^, ^6^ Given the association between chest compression depth and OHCA outcomes,^7^ this depth range has been incorporated into CPR training programs, CPR feedback devices and dispatcher-assisted CPR (DACPR) protocols.^8^, ^9^, ^10^, ^11^

However, previous studies indicate that both laypersons and health care providers often fail to achieve chest compressions within the recommended depth range.^12^, ^13^, ^14^ Although interventions such as real-time feedback devices, updated DACPR terminology, and video-assisted CPR have been introduced to improve prehospital CPR quality,^7^, ^8^, ^15^ bystander CPR during OHCA relies heavily on audio-only instructions provided by dispatchers.

During DACPR, dispatchers instruct bystanders to perform CPR prior to emergency medical services arrival when OHCA is suspected.^9^ However, studies have shown that laypersons frequently struggle to achieve target chest compression depth when instructed using specific numeric values.^8^, ^16^, ^17^ In addition, research suggests that simplified verbal instructions by dispatchers, such as “push as hard as you can,” could result in more effective CPR performance than specifying numeric chest compression depth targets.^8^, ^16^, ^17^

Performing chest compressions to a specific numeric depth, as instructed during DACPR, requires accurate estimation of a given length, translation of that estimation into vertical depth, and application of appropriate force to achieve the instructed depth. Limitations in the ability to accurately estimate chest compression depth may represent a systemic barrier to bystander CPR effectiveness, yet limited evidence exists regarding laypersons' ability to translate lengths into vertical depth and chest compression depths. Therefore, this study aimed to investigate the accuracy of laypersons in estimating a length of 50 mm, translating the estimated length into vertical depth, and performing chest compressions to a 50 mm depth. We hypothesized that significant cognitive limitations would be observed, highlighting a systemic barrier to effective DACPR.

## Methods

### Study design and population

This prospective observational study was conducted from September 1, 2024, to January 31, 2025, in Seoul, South Korea. We enrolled adult laypersons without CPR training within the past two years. Participants were recruited through participating hospitals' websites and affiliated clinical trial recruitment platforms. Participants received USD 20 in compensation upon completion of the study protocol.

### Study protocol

After enrolment, participants visited the study site and completed the following steps.

Step 1: Participants were asked to draw a 50 mm horizontal line on an A4 sheet of paper using a pen. After completion, participants were asked to press a vertical depth measurement plate vertically to what participants estimated to be a depth of 50 mm without support (Supplementary Fig. 1). The depth measurement plate was intentionally designed with minimal resistance to minimize the influence of tactile feedback on depth perception. The device was placed on a table and participants were asked to press the plate down with a single hand while standing beside the device. Subsequently, participants performed five chest compressions on a CPR training manikin to the estimated depth of 50 mm. Chest compression was performed on BT-SEEM-Air CPR training manikin (BT Inc., Gyeonggi-do, South Korea), which provides real-time depth measurements up to 80 mm. The manikin was placed on the floor, and participants were instructed to kneel beside the manikin and perform chest compressions using both hands.

Step 2: Participants were presented with a horizontal reference line of 50 mm printed on paper. While viewing the visual reference, participants pressed the vertical depth measurement plate and performed five chest compressions on the CPR manikin, both targeting an estimated depth of 50 mm.

### Outcomes

The primary outcome was the accuracy of vertical depth estimation without provision of visual reference. Secondary outcomes included: accuracy of horizontal length estimation, accuracy of chest compression depth on the manikin before viewing visual reference, accuracy of depth estimation after viewing visual reference and accuracy of chest compression depth on the manikin after viewing visual reference. Accuracy was quantified as the mean difference of estimations from the gold standard of 50 mm. An estimation was classified as an overestimation if it exceeded 50 mm and an underestimation if it failed to reach 50 mm. In addition, the proportions of estimations within an acceptable range of ±5 mm (i.e., 45–55 mm) for each task were calculated.

### Statistical analysis

Based on previous research indicating approximately 35 % of laypersons could accurately estimate horizontal lengths within target,^18^ a minimum of 88 participants were required to estimate this proportion with a 95 % confidence interval and a 10 % margin of error. Accounting for potential data loss, mechanical errors, and participant dropout, the final recruitment target was set at 100 participants.

Continuous variables were reported as means with standard deviations (SD) or medians with interquartile ranges (IQR) as appropriate. Categorical variables were reported as numbers with percentages. Chest compression depth was calculated as the average of five consecutive compressions on the CPR training manikin. One-sample t-tests or Wilcoxon signed-rank tests were used to compare accuracy of estimations against the gold standard. Changes in accuracy before and after provision of visual references were assessed using Wilcoxon signed-rank test and McNemar's test. Correlations between horizontal length estimation, vertical depth estimation, and chest compression depths were analyzed using Spearman's correlation. A two-tailed *p*-value of <0.05 was considered statistically significant. Statistical analysis was performed using Stata, Version 16 (StataCorp, College Station, TX).

### Ethics statement

The study was approved by the institutional review board of the participating institution (IRB No. 10-2024-49). Informed consent was obtained from all participants.

### Trial registration

This study was registered at ClinicalTrials.gov (Identifier: NCT06552299). The registered protocol included both layperson and healthcare provider groups (N = 100 each). This manuscript presents the results for the layperson group. Findings from the healthcare provider group are reserved for a separate publication.

## Results

A total of 100 laypersons were enrolled in the study (Table 1). The mean age was 45.5 years (SD 11.2), and 35 participants (35.0 %) were male. Only fifteen participants (15.0 %) had received CPR training prior to study participation. None had CPR training within the past two years.

**Table 1:** Baseline characteristics of study participants.

	N (%) or Mean (SD)
Total	100 (100.0)
Male sex	35 (35.0)
Age (yr)	45.5 (11.2)
Cardiopulmonary resuscitation training experience	15 (15.0)
Height (cm)	165.5 (9.0)
Weight (kg)	64.7 (12.6)
Body Mass Index	23.5 (3.2)

SD, standard deviation.

Primary analysis results are shown in Table 2. Participants significantly underestimated horizontal length, with a mean difference from 50 mm of −3.5 mm (SD 16.6, *p* = 0.036). Only 25.0 % (95 % CI: 16.9 %–34.7 %) of estimations fell within the acceptable range of 45–55 mm.

**Table 2:** Accuracy of length, depth and chest compression depth estimations before and after provision of visual reference.

	Mean	Difference from 50 mm		Estimations within acceptable range (45–55 mm)		
		Mean Difference (SD)	*P*-value	Proportion (95 % CI) (%)	Improvement after providing visual reference	
					Risk difference (95 % CI) (%)	*P*-value
Estimated horizontal length (mm)	46.5	−3.5 (16.6)	0.036	25.0 (16.9–34.7)		
Estimated vertical depth before providing visual reference (mm)	54.9	4.9 (19.4)	0.044	26.0 (17.7–35.7)	6.0 (−6.6–18.6)	0.451
Estimated vertical depth after providing visual reference (mm)	51.4	1.4 (17.4)	0.656	32.0 (23.0–42.1)		
Chest compression depth before providing visual reference (mm)	44.5	−5.5 (10.7)	<0.001	35.0 (25.7–45.2)	1.0 (−12.3–14.3)	1.000
Chest compression depth after providing visual reference (mm)	44.7	−5.3 (11.0)	<0.001	36.0 (26.6–46.2)		

SD, standard deviation; CI, Confidence interval.

**Fig. 1:**
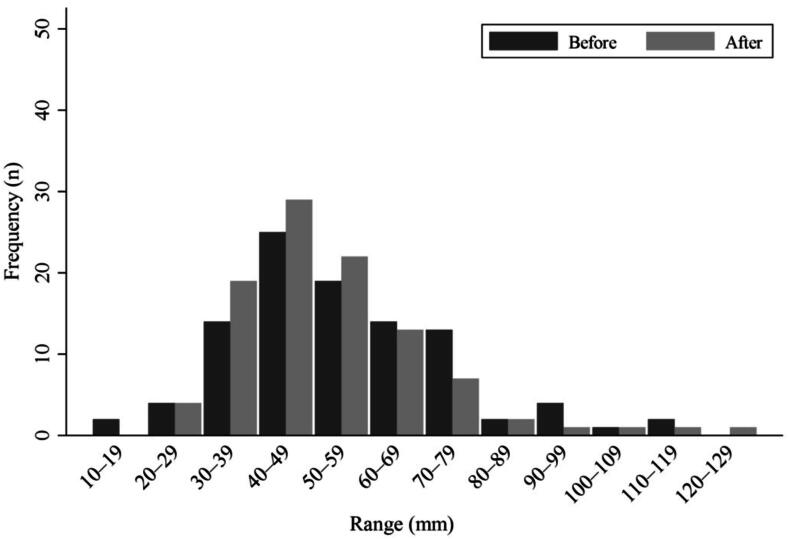
Distribution of vertical depth estimations before and after provision of visual reference, categorized by depth range.

Participants significantly overestimated vertical depth, with mean difference from 50 mm of +4.9 mm (SD 19.4, *p* = 0.044). Only 26.0 % (95 % CI: 17.7 %–35.7 %) of estimations were within the acceptable range. After provision of a visual reference, vertical depth estimation improved slightly; however, the improvement was not statistically significant for either mean depth (p = 0.123) or the proportion within the acceptable range (*p* = 0.451). Fig. 1 illustrates the distribution of vertical depth estimates before and after provision of visual reference.

**Fig. 2:**
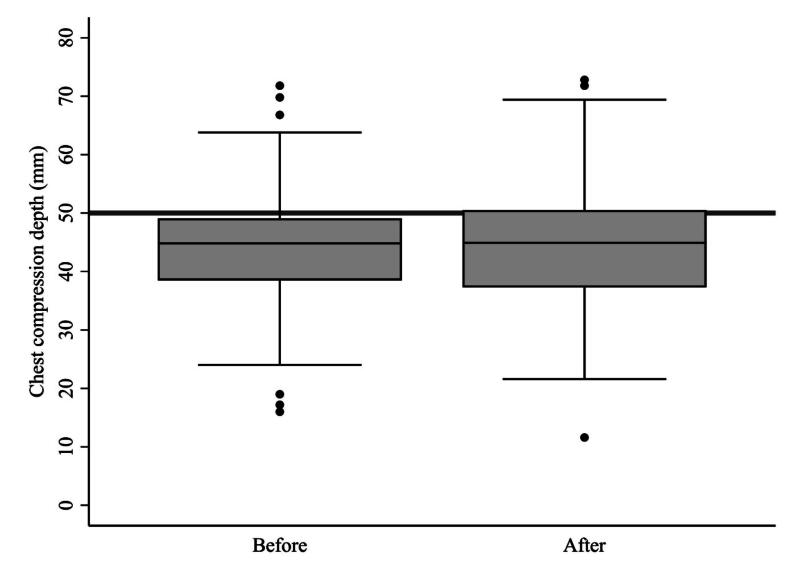
Box plot of chest compression depth before and after provision of visual reference. Boxes represent the median and interquartile range; whiskers indicate the full range excluding outliers. The horizontal line at 50 mm indicates the gold standard.

Chest compression depth was significantly underestimated both before (mean 44.5 mm, SD 10.7, *p* < 0.001) and after (mean 44.7 mm, SD 11.0, *p* < 0.001) viewing the visual reference (Fig. 2). The improvement in chest compression depth after provision of the visual reference was not statistically significant for mean depth (*p* = 0.548) or for the proportion within the acceptable range (*p* = 1.000).

**Fig. 3:**
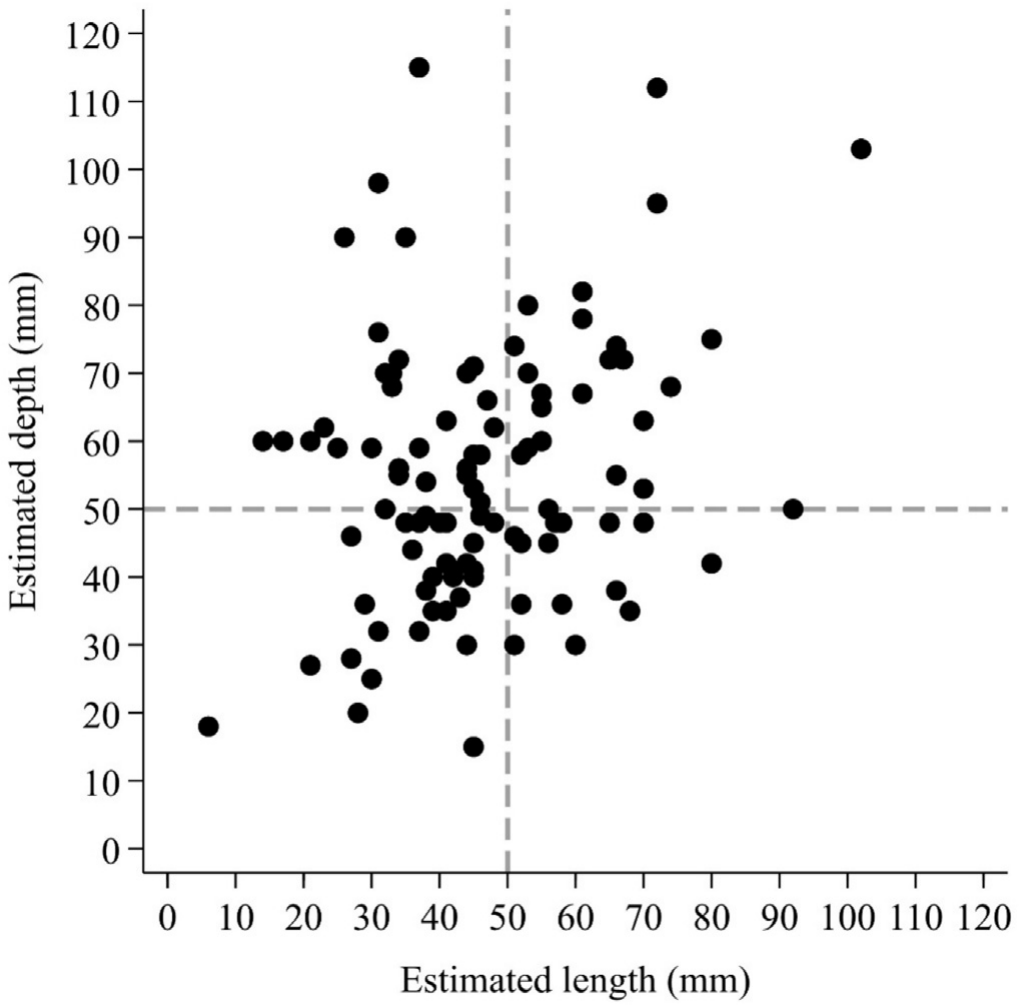
Scatterplot of length estimations vs. vertical depth estimations before provision of visual reference. Each point represents a participant’s estimations in horizontal length (x-axis) and vertical depth (y-axis). Dashed lines indicate the gold standard of 50 mm for both axes.

Results of the correlation analysis between variables prior to visual reference are presented in Table 3. A statistically significant but weak correlation was observed between vertical depth estimation and chest compression depth (*p* = 0.019). Horizontal length estimation was not significantly correlated with vertical depth estimation (*p* = 0.075; Fig. 3) or with chest compression depth (*p* = 0.093).

**Table 3:** Correlation between length, depth and chest compression depth estimations before provision of visual reference.

	Spearman’s ρ	*P*-value
Estimated horizontal length vs. estimated vertical depth	0.179	0.075
Estimated horizontal length vs. chest compression depth	0.169	0.093
Estimated vertical depth vs. chest compression depth	0.235	0.019

## Discussion

This study demonstrates that laypersons have significant difficulty accurately estimating 50 mm in horizontal length, vertical depth, and chest compression depth. In addition, providing a visual reference did not significantly improve estimation accuracy or chest compression performance. Correlations between horizontal length, vertical depth, and chest compression depth were negligible to weak, indicating limited translation among these estimations.

Previous studies have reported that both laypersons and health care providers have difficulty performing chest compressions within the CPR guideline recommended depth range.^12^, ^13^, ^14^ Suboptimal bystander CPR performance has been attributed to multiple factors, including inadequate training, anxiety, advanced age, limited physical fitness, fatigue, resuscitation environment, and poor compliance with dispatcher instructions, as well as the inherent difficulty of intuitively perceiving length and depth.^18^, ^19^, ^20^, ^21^, ^22^, ^23^, ^24^ Tulder et al. reported that both laypersons and health care providers struggle to estimate horizontal distances, suggesting that impaired spatial estimation could contribute to inadequate compression depth.^18^ In addition, several studies have reported that health care providers’ visual assessment of CPR quality is often inaccurate.^25^, ^26^, ^27^

DACPR protocols that instruct bystanders without previous CPR training to perform chest compressions to a specific depth assume that bystanders have an accurate perception of how long the specific depth is and can accurately translate the length to vertical chest compression depth. However, our findings indicate that laypersons have a limited perception of horizontal length and have difficulty in estimating vertical depth even when provided with reference. In addition, correlation analysis suggests that there are limitations in translating horizontal length into vertical depth and chest compression depth. These findings suggest that current DACPR protocols relying on numeric compression depth instructions may face inherent cognitive limitations at the lay rescuer level, reducing their effectiveness. Although previous studies have proposed the use of anatomical landmarks to guide rescuers in achieving adequate compression depth,^28^, ^29^ the low accuracy of depth estimation observed after visual reference provision in our study suggests that the effectiveness of visual references in guiding CPR could be limited.

Our findings align with prior research demonstrating that simplified CPR instructions, such as “push as hard as you can,” resulted in higher quality and faster initiation of chest compressions than when specific numeric depth instructions were provided.^8^, ^16^, ^17^, ^30^ Although concerns have been raised regarding potential for injuries associated with chest compressions to excessive depths, previous studies suggest that laypersons tend to perform shallow chest compressions even when instructed to “push hard and fast,” and that compression-related injuries are rarely fatal.^8^, ^31^ Additionally, concerns have been raised that providing upper limits in chest compression instructions could unintentionally discourage rescuers from delivering compressions of adequate depth, as lay rescuers have limited ability to distinguish between lower and upper thresholds or to adjust their compressions accordingly.^18^, ^32^, ^33^

While real-time feedback devices have been utilized to optimize CPR quality, their use is limited by availability to bystander on site when needed.^7^, ^26^ Video assistance during DACPR has been implemented and enables dispatchers to visually assess bystander CPR performance; however, challenges remain regarding the transition from audio to video calls and the reliability of visually estimating CPR quality.^15^, ^34^ Given these limitations, DACPR system improvements should prioritize simplified, action-based dispatcher instructions and widespread training initiatives that emphasize deliberate hands-on practice and mastery learning to develop adequate depth perception.

The study has several limitations. First, our definition of an acceptable range was set at 45–55 mm, and different threshold ranges could yield different findings. Second, our reference target of 50 mm was selected to reflect the lower bound of the guideline-recommended chest compression depth. While using a different target could have changed the mean estimations, the study's primary finding regarding the inaccuracy of these estimations would likely remain unchanged. Additionally, estimation accuracy could have been influenced by external factors, including paper size, measurement plate resistance, and manikin compliance. Familiarity with the manikin may also have affected performance. While the vertical depth measurement plate was designed to assess the ability to estimate vertical depth, it did not replicate chest compressions on a human thorax; to mitigate the limitation, participants also performed chest compressions on a CPR training manikin. Furthermore, the measurement of chest compression depth from only the initial five compressions represents a significant limitation, as it does not account for the dynamic nature of CPR, rescuer fatigue, or the potential for performance adjustments that could occur during a more sustained period. Finally, this was a simulation-based study conducted in a controlled environment, which may not generalize to real-world out-of-hospital settings.

## Conclusions

Laypersons demonstrated significant limitations in estimating horizontal length, vertical depth, and chest compression depth. Providing a visual reference did not significantly improve performance, and horizontal length, vertical depth, and chest compression depth estimations showed negligible to weak correlations. These findings suggest that reliance on numeric compression depth instructions in DACPR protocols may represent a systemic barrier to effective bystander CPR delivery.

## Declaration of Generative AI and AI-Assisted Technologies in the Writing Process

During the preparation of this work, the authors used ChatGPT (OpenAI, GPT-4) to refine the language and improve readability. After using this tool, the authors reviewed and edited the content as needed and take full responsibility for the content of this publication.

## CRediT authorship contribution statement

**Min Woo Kim:** Writing – original draft, Visualization, Investigation, Formal analysis, Data curation. **Stephen Gyung Won Lee:** Writing – review & editing, Validation, Supervision, Software, Resources, Project administration, Methodology, Funding acquisition, Formal analysis, Conceptualization. **Tae Han Kim:** Writing – review & editing, Methodology, Investigation, Data curation. **Yoon Ha Joo:** Writing – review & editing, Resources, Methodology, Investigation, Data curation. **Ki Jeong Hong:** Writing – review & editing, Supervision, Resources, Methodology.

## Funding

This study was supported by research funding from the Seoul Metropolitan Government 10.13039/501100002551Seoul National University (SMG-SNU) Boramae Medical Center (04-2024-0012).

## Declaration of competing interest

The authors declare that they have no known competing financial interests or personal relationships that could have appeared to influence the work reported in this paper.
