# Supplementary material for: Cognitive limitations in depth estimation for dispatcher-assisted cardiopulmonary resuscitation: a prospective simulation study
Source: Resusc Plus. 2025 Sep 11;26:101093. doi: 10.1016/j.resplu.2025.101093 (PMC12492283; doi:10.1016/j.resplu.2025.101093)

**Supplementary materials**

**Supplementary Figure 1.** Vertical depth measurement plate (A) Lateral view of the device showing a graduated scale in centimeters (B) Overhead view demonstrating participant hand placement during vertical depth estimation. Participants were blinded to the scale markings during all measurements.


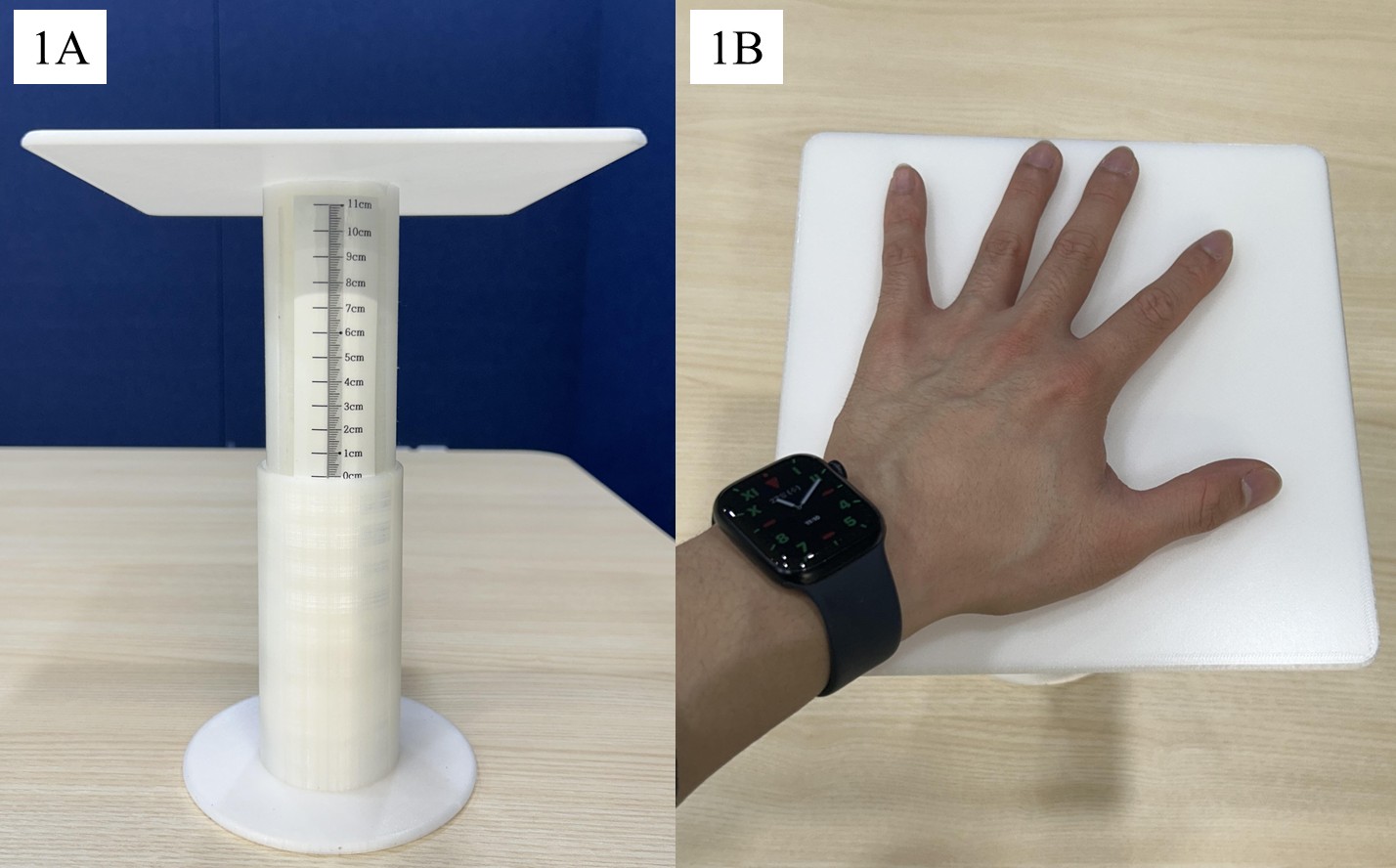

Supplement: Supplementary Data 1 [file mmc1.docx]
